# A Look at Primary and Secondary Prevention in the Elderly: The Two Sides of the Same Coin

**DOI:** 10.3390/jcm13154350

**Published:** 2024-07-25

**Authors:** Maurizio Giuseppe Abrignani, Fabiana Lucà, Vincenzo Abrignani, Giuseppe Pelaggi, Alessandro Aiello, Furio Colivicchi, Francesco Fattirolli, Michele Massimo Gulizia, Federico Nardi, Paolo Giuseppe Pino, Iris Parrini, Carmelo Massimiliano Rao

**Affiliations:** 1 O.U. Cardiology-ICCU, P. Borsellino Hospital, ASP Trapani, 91025 Marsala, Italy; 2 O.U. Interventional Cardiology, Bianchi Melacrino Morelli Hospital, 89124 Reggio Calabria, Italy; fabiana.luca92@gmail.com (F.L.);; 3 Internal Medicine and Stroke Care Ward, Department of Health Promotion, Mother and Child Care, Internal Medicine and Medical Specialties, University of Palermo, 90141 Palermo, Italy; 4 Cardiology Division, San Filippo Neri Hospital, 00135 Rome, Italy; 5 Department of Experimental and Clinical Medicine, Careggi University Hospital, University of Florence, 50121 Firenze, Italy; 6 O.U. Cardiology, Garibaldi-Nesima Hospital, ARNAS Garibaldi, 95123 Catania, Italy; 7 O.U. Cardiology, Santo Spirito Hospital, 15033 Casale Monferrato, Italy; federico.nardi1@gmail.com; 8 Heart Diagnostics Division, A.O. S. Camillo-Forlanini, 00152 Rome, Italy; 9 Cardiology Department, Mauriziano Umberto I Hospital, 10128 Turin, Italy

**Keywords:** aging, cardiovascular prevention, comorbidity, elderly, risk factors

## Abstract

The global population is experiencing an aging trend; however, this increased longevity is not necessarily accompanied by improved health in older age. A significant consequence of this demographic shift is the rising prevalence of multiple chronic illnesses, posing challenges to healthcare systems worldwide. Aging is a major risk factor for multimorbidity, which marks a progressive decline in resilience and a dysregulation of multisystem homeostasis. Cardiovascular risk factors, along with aging and comorbidities, play a critical role in the development of heart disease. Among comorbidities, age itself stands out as one of the most significant risk factors for cardiovascular disease, with its prevalence and incidence notably increasing in the elderly population. However, elderly individuals, especially those who are frail and have multiple comorbidities, are under-represented in primary and secondary prevention trials aimed at addressing traditional cardiovascular risk factors, such as hypercholesterolemia, diabetes mellitus, and hypertension. There are concerns regarding the optimal intensity of treatment, taking into account tolerability and the risk of drug interactions. Additionally, uncertainty persists regarding therapeutic targets across different age groups. This article provides an overview of the relationship between aging and cardiovascular disease, highlighting various cardiovascular prevention issues in the elderly population.

## 1. Introduction

Aging is a biological parameter characterized by the gradual deterioration of the organism until eventual death. Elderly individuals are typically defined as those surpassing a predetermined age threshold, often determined by retirement age [1,2,3]. The World Health Organization delineates the elderly population into distinct categories: those aged 60–74 are classified as the elderly and individuals aged 75–90 and those over 90 as the oldest-old. Alternatively, geriatricians prefer to classify this demographic as “young elderly” (65–74 years old), “elderly” (75–84 years old), and “oldest-old” or long-lived individuals (over 85 years old). With advancing age, there is also a higher proportion of females [1,2,3,4,5]. The duration of human life is influenced by both environmental and genetic factors that interact with each other. Certain genetic variations, such as the absence of the ε4 allele of the APOE polymorphism, have been linked to increased longevity. The antagonistic pleiotropy theory posits that aging is driven by genes that have beneficial effects in youth, thus conferring evolutionary advantages, but become detrimental at older ages [6]. Ultimately, adaptive mechanisms in individuals with unfavorable genetic backgrounds who manage to survive until old age aid them in navigating the challenges associated with aging. On the environmental front, over the past two centuries, health standards have improved significantly owing to enhancements in hygienic and nutritional conditions, advancements in medical care (for both infectious and chronic diseases), and the efficacy of healthcare systems. Consequently, the resulting decrease in infant and overall mortality rates has enhanced survival prospects across all age groups [7].

Hence, the global life expectancy at birth has witnessed a notable increase [8], particularly pronounced among women and in more developed regions. Additionally, there is a noteworthy trend toward progressive aging within the elderly population. Individuals reaching 65 can anticipate an additional 16 years, on average, of life expectancy, with a more pronounced increase compared to life expectancy at birth.

The demographic landscape is experiencing significant shifts in the age structure [9,10,11], trending toward a “demographic winter”, characterized by a declining number of children and a rising proportion of elderly individuals. This demographic transition process is attributed to declining birth rates and the “baby boom” cohort aging. The age pyramid is the most effective graphical representation to illustrate this phenomenon [11]. The traditional triangular profile has transformed, characterized by diminishing bases attributable to low birth rates and continuously expanding tops resulting from increased life expectancy, leading to a rectangular or even trapezoidal shape. The present age structure is heavily influenced by historical trends and serves as a foundation for future projections. The notably large cohorts currently occupying middle-age brackets will gradually shift upward. This phenomenon is anticipated to yield substantial health and socioeconomic implications [12].

Health interventions are mandatory for improving the prognosis and quality of life (QoL) in elderly people. Research on cardiac aging is now becoming a pivotal phase of paramount importance for clinical medicine and social welfare advancement. However, clinical trials have typically involved a limited number of elderly patients. Consequently, existing clinical practice guidelines lack specific recommendations on cardiovascular (CV) prevention tailored to the elderly. This review aims to evaluate prevention recommendations for elderly patients with CV risk, encompassing objectives, medical and device treatment options, and cardiac rehabilitation programs.

## 2. Chronological Age and Biological Age: Assessing the Complexity and Frailty of the Elderly

Aging is a complex phenomenon influenced by multiple factors and characterized by a gradual deterioration in the functionality of molecules, cells, and organs. This decline leads to a reduction in various functional capacities, including physical, mental, and sensory abilities. Moreover, aging is associated with an increased prevalence of comorbidities due to the accumulation of risk factors, a homeostasis imbalance, and resilience mechanisms [6,9].

The prevalence of chronic diseases is notably high among the elderly population, often accompanied by comorbidities, which heightens the risk of interactions between different pathologies and pharmacological interventions. Globally, CVD stands as the primary contributor to morbidity and mortality among older individuals. The incidence of CV events rises notably after the age of 65 in men and after 75 in women. Several CV factors, including blood glucose levels and systolic blood pressure, demonstrate a progressive increase with advancing age. In contrast, other factors, such as body weight, cholesterol levels, diastolic blood pressure, and heart rate, tend to decline [9].

The weight of CV risk factors is relatively minor, primarily due to the survival effect, wherein carriers of such factors tend to experience premature mortality. Moreover, age emerges as one of the most significant determinants in the onset of diseases. While age per se does not directly induce cardiovascular damage, it signifies a critical juncture wherein cardioprotective systems decline, leaving the heart vulnerable to prolonged exposure to detrimental factors like comorbidities and environmental influences.

Advancing age precipitates structural and functional alterations in the cardiovascular system, leading to a diminished capacity for a stress response. Consequently, mortality rates and the incidence of cardiovascular diseases among the elderly exhibit a declining trend, attributable to factors such as reduced smoking prevalence and improved detection and management of hypertension, dyslipidemia, and diabetes. However, this decline correlates with an increase in the occurrence of neoplasms. Furthermore, while gains have been made in prolonging the lifespan, similar advancements in enhancing the quality of life have not been commensurate.

Aging and concurrent comorbidities significantly elevate the risk of disability, characterized by limitations or the inability to independently perform basic or advanced activities of daily living, thereby diminishing the overall quality of life. Surveys indicate that over a third of individuals aged 75 and above face challenges in managing finances, taking medication, or using the telephone, while nearly half experience difficulties in activities like meal preparation, grocery shopping, or light household chores. Notably, between 1990 and 2013, while life expectancy steadily increased, healthy life expectancy improved slower, and disability life expectancy exhibited a notable decline of 27% [13].

It is crucial to recognize that aging itself does not inevitably predispose individuals to illness. Treating the elderly population as a homogeneous group would oversimplify matters, disregarding the significant diversity in their physical and mental health statuses.

Prioritizing a healthy lifestyle and fostering robust social connections can significantly contribute to maintaining optimal psychophysical capacities over an extended period. This underscores the importance of acknowledging the multifaceted nature of aging and adopting holistic approaches to promote well-being in older adults [3].

Another important consequence of demographic changes is the increase in the global mortality rate, as the incidence of non-communicable diseases rises with age, and the elderly are at a higher risk of death [14].

About 80% of deaths from CVD are attributed to those over 65 [11].

The aging phenomenon is reshaping the social fabric of numerous countries, exerting profound effects on the sustainability of healthcare and pension systems. This demographic shift presents a complex interplay of challenges and opportunities. Conversely, the escalating number of elderly individuals grappling with chronic comorbidities and disabilities underscores the pressing need for expanded social and healthcare provisions.

Many laboratory investigations aimed at screening and monitoring classic CV risk factors may hold diminished relevance in geriatric populations. This is either because these risk factors have already precipitated their deleterious effects or because, given life expectancy considerations, their impact may be limited. Consequently, allocating resources judiciously becomes imperative.

Moreover, healthcare expenditures tend to escalate significantly during the final years of life and in proximity to death. This underscores the necessity for strategic resource allocation and policy interventions to optimize the efficiency and effectiveness of healthcare delivery systems, particularly in the context of an aging population [14,15]. 

The concept of “biological age” typically encompasses the “pathological” aging process, which reflects the cumulative impact of comorbidities and the decline in the functional reserve associated with disease states influenced by various genetic susceptibilities. Despite its theoretical significance, the practical utility of “biological age” as an independent prognostic parameter remains poorly defined, primarily due to its measurement and quantification challenges. Consequently, it is often excluded from conventional risk stratification methodologies.

One prevailing framework for understanding biological age involves three fundamental determinants: chronological age, the peak of functional capacity (largely determined by genetics), and the rate of aging, notably influenced by environmental factors and lifestyle choices. This multifaceted approach acknowledges the complex interplay between intrinsic biological processes and external influences in shaping an individual’s aging trajectory [13].

For many patients, the designation of “complex” aims to convey the intricate nature of required treatments and the presence of multiple comorbidities. However, consensus on precisely defining the “complex elderly” remains elusive. This categorization should encompass a wide array of variables beyond medical conditions. These may include disease complications, as well as the direct and collateral effects of treatments.

Moreover, a comprehensive understanding of complexity in the elderly should extend beyond biological factors to encompass psychological, socio-economic, cultural, and environmental aspects. Social vulnerability, reflecting an individual’s susceptibility to adverse health outcomes due to societal circumstances, is also integral to this characterization.

Recognizing and addressing this multidimensional complexity is crucial for developing tailored care approaches that adequately meet the diverse needs of complex elderly patients [16].

Another term frequently used for elderly subjects or patients is “fragile”. Frailty and comorbidity can be coexisting but also independent conditions. Therefore, there is a high likelihood of a frail elderly person without comorbidity, a multi-morbid non-fragile complex patient, and a non-necessarily comorbid disabled person. Frailty represents the global phenotype of a physiologically reduced homeostatic reserve and greater vulnerability to stressors, exposing the individual to an increased risk of disability or death [17]. The “frailty phenotype” derived from the Cardiovascular Health Study and the Canadian Study of Health and Aging consists of two models. The first model includes five domains: unintentional weight loss, muscle strength measured with the handgrip, fatigue, walking speed, and amount of habitual physical activity [18].

In the second model, a frailty index was formulated using 70 functional and clinical parameters examining physical, cognitive, and activities-of-daily-living abilities. Regular assessment of frailty in participants involved in clinical drug trials would enhance the understanding of treatment effects on the frail population and facilitate the identification of individuals who stand to gain more or less from the intervention [19]. Data from 17 studies assessing frailty demonstrated that the effect of frailty on treatment efficacy is not consistent [20].

While various trials have demonstrated diminished treatment effects in frail patients, the majority indicate that the efficacy of the treatment remains unaffected by frailty. In some cases, the benefits of the treatment were found to be more pronounced in frailer patients [20].

Social support and caregiver support are recognized as pivotal factors for secondary prevention among patients with CVD [21].

Comorbidity, which is characterized by the concurrent presence of two or more pathologies within a single patient, tends to escalate with age and is linked with an elevated risk of mortality, rehospitalization, disability, and decline in the quality of life. This risk surpasses that associated with individual pathologies alone. The significant prevalence of comorbidity poses challenges in extrapolating trial outcomes to the geriatric population, as studies often systematically exclude elderly individuals with multiple morbidities. Moreover, intervention studies typically limit the reporting of CV comorbidity to a mere enumeration of diseases, without delving into their specific impacts [22]. Various tools have been developed for comorbidity assessment, including Greenfield’s Index of Coexisting Disease and the Geriatric Index of Comorbidity [23].

In the past few years, physical evaluation has become of paramount importance [17]. The Short Physical Performance Battery (SPPB) [24] has garnered particular attention. This assessment, which trained professionals can complete in a few minutes, demonstrates an independent predictive capacity for outcomes in elderly patients with HF, alongside their New York Heart Association (NYHA) class. The SPPB evaluates three key domains: balance, lower limb strength, and walking speed. Notably, walking speed has emerged as a particularly potent prognostic indicator across numerous CV pathologies in the elderly.

Specific tools are required to assess cognitive decline, such as the Mini-Cog Test, a straightforward clinical screening test involving the repetition of three words and drawing a clock set to a specified time [25].

Functional decline is influenced by a myriad of factors, including comorbidity, sarcopenia, inflammation, cognitive status, depression, balance, and sleep disorders, which intricately intertwine. However, most major studies investigating cardiovascular therapies tend to focus on outcomes like mortality, recurrence, and hospitalizations, often neglecting to evaluate the risks associated with functional loss. Many elderly prioritize preserving basic activities of daily living and maintaining independence as their primary goals. Thus, a comprehensive assessment encompassing multiple dimensions and involving a multidisciplinary team, including the patient and their caregivers, must accurately identify the patient’s profile and determine the most suitable care pathway, while optimizing resource use [26].

**Figure 1:**
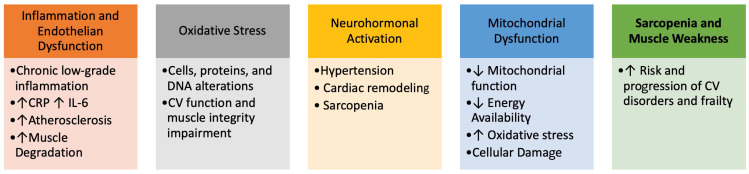
Interconnections between frailty and Cardiovascular Disorders.

CVD and frailty, indeed, share a close and interdependent relationship, both being influenced by similar physiological and pathological processes. Several key mechanisms underline their interconnection (Figure 1).

Several underlying pathophysiological mechanisms contribute to the onset of frailty, including cognitive decline, physical inactivity, poor nutrition, and insufficient social support. These risk factors present multiple avenues for intervention strategies designed to prevent, ameliorate, or reverse the progression of frailty syndrome, particularly in the context of CVD.

The diagnosis of frailty is based on the presence of five components: unintentional weight loss, weakness, poor endurance and energy, slowness, and low levels of physical activity. Unintentional weight loss, indicative of sarcopenia, is a contributing factor in the development of frailty.

Cognitive dysfunction similarly increases vulnerability, leading many researchers to suggest incorporating cognitive aspects into the definition of frailty.

**Figure 2:**
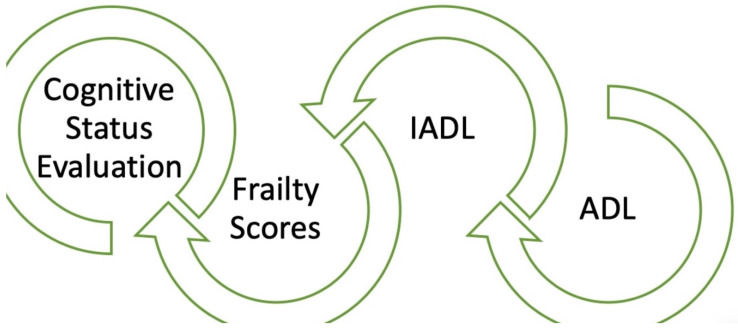
Combined evaluation of elderly people: cardiogeriatricteam’s tools.

Measuring frailty is crucial for predicting outcomes and devising suitable treatment plans for patients with CVD. A variety of tools have been developed to assess frailty; some are specifically designed to evaluate physical frailty, while others encompass cognitive and psychosocial dimensions (Figure 2). Intervention strategies may be categorized into primary and secondary prevention.

Finally, it should be noted that the evaluation of elderly people (ADL, IADL, degree of frailty, evaluation of cognition) (Figure 2) should be carried out either by personnel specialized in geriatrics or by geriatric cardiologists. It is easy, in fact, to obtain false results in this field, as communication with elderly people is not easy.

## 3. Heart Changes in the Elderly

In old age, heightened sympathetic activity leads to the overstimulation of adrenergic receptors, resulting in adverse alterations within the associated signaling pathways, including progressive myocardial remodeling [27,28]. These changes may include decreased receptor quantity and reduced efficiency of downstream effectors [28]. Imaging techniques, particularly echocardiography, can identify age-related CV alterations beyond pathological conditions.

Changes in the valves within the left heart chambers are notably more pronounced. The aortic cusps undergo thickening and develop calcifications, while the annulus also thickens, promoting calcium deposition. Despite the observed thickening of the valves on echocardiography, their dynamics are preserved and do not result in significant hemodynamically relevant obstruction or regurgitation. Although most cases of senile sclerosis do not progress to stenosis, increased mechanical stress at the base of the cusp fosters the formation of calcifications, which, in turn, diminish valve dynamics and can lead to varying degrees of obstruction, including severe stenosis [29].

Mitral leaflets may show nodular sclerosis and limited or complete calcification of the annulus, particularly in women [30,31].

A variant of valve calcification is caseous calcification, which can sometimes mimic a neoplasm, referred to as tumor-like annular calcification. While the echocardiographic appearance is often typical, magnetic resonance imaging may be necessary for differential diagnosis. Annular calcification is frequently observed in elderly patients with long-standing hypertension, varying degrees of renal insufficiency, and obesity, and it may be associated with conditions such as atrial fibrillation, arterial thromboembolism, and, rarely, endocarditis. The frequent coexistence of mitral and aortic valve apparatus calcification in the elderly, forming a continuous anatomical entity, led Roberts in 1986 to introduce the term “senile calcific cardiac syndrome”, which is associated with an unfavorable prognosis.

The size of the aortic root increases by approximately 6% from the fourth to the eighth decade of life. The left ventricular volume remains stable with age, in contrast to the increased wall thickness and mass. Elderly individuals, particularly those with arterial hypertension (AH), often exhibit increased thickness of the basal septum, termed “sigmoid” or “senile”.

Systolic function, as assessed by the ejection fraction, and systolic output, measured by integrated two-dimensional Doppler, remain constant with age. However, diastolic function often demonstrates a reduced early diastolic wave and an increased late diastolic wave in the elderly, reflecting decreased left ventricular elasticity. Mild left atrial enlargement in the elderly is suggested to be due to increased atrial contraction. Heart failure with a preserved ejection fraction (HFpEF) is prevalent in the elderly, particularly among women; echocardiography is instrumental in identifying underlying diastolic dysfunction [32,33].

Another etiology of HF is systemic light-chain amyloidosis, characterized by increased myocardial thickness and an initially preserved ejection fraction. Global longitudinal strain (GLS) analysis is particularly useful as it can reveal normal strain values in the apical region, either as an absolute value or relative to other segments. Furthermore, amyloidosis has been linked to aortic valve stenosis.

In the elderly, alternative imaging modalities beyond echocardiography are seldom used, with echocardiography remaining the primary diagnostic tool. However, additional imaging techniques may be warranted based on echocardiographic findings and clinical requirements. CT angiography is often preferred for suspected aortic vessel pathology due to its high spatial resolution, enabling visualization of the thoracic and abdominal aorta, as well as its branching vessels. In suspected amyloidosis cases, gadolinium-enhanced MRI is valuable for diagnosis, using the patchy distribution of late gadolinium enhancement as a diagnostic criterion. Both radiological techniques involve using contrast agents, necessitating caution in elderly patients who may present with varying degrees of renal insufficiency. Technetium pyrophosphate scintigraphy is also used to diagnose amyloidosis, highlighting potential cardiac accumulation of activity.

## 4. Cardiovascular Prevention in the Elderly

Risk prediction models, equations, and charts are pivotal tools in the primary prevention of CVD, aiding in risk management decisions. However, their utility in older individuals remains uncertain. The elderly population differs from the general population in terms of model algorithms and the strength of association between predictors and outcomes, leading to attenuated prediction performance of these models [34].

Age emerges as the primary driver of CVD risk, and the estimated 10-year CVD risk exceeds conventional risk thresholds for almost all individuals over 70 years old [34].

Additionally, the cumulative advantage of treatment, measured by the duration of CVD-free life gained, diminishes with advancing age. This emphasizes the need for judicious evaluation and potentially customized strategies to apply risk prediction models among older individuals. While preventive healthcare measures have alleviated the burden of chronic ailments among younger cohorts, evidence supporting their efficacy in enhancing health outcomes among older populations remains limited.

**Figure 3:**
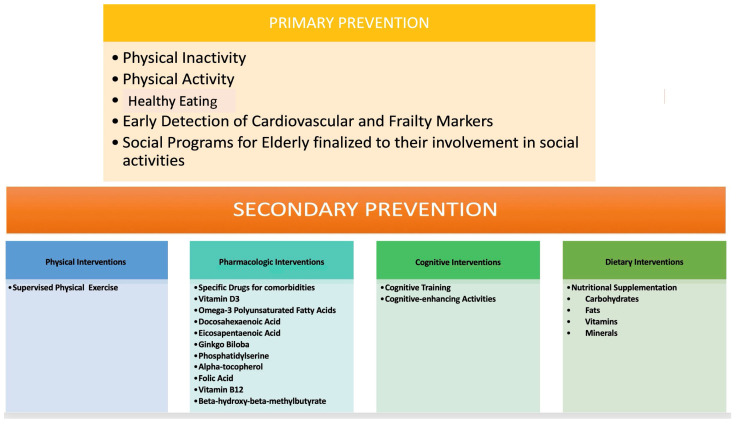
CV prevention therapy.

The primary prevention of CV events in the elderly poses a pertinent challenge, given the dearth of evidence regarding safe and effective therapeutic interventions alongside associated costs. Therefore, it is imperative to optimize the quality of life for patients and extend their healthy lifespan by selecting optimal treatments tailored to individual needs. It is crucial to bear in mind that elderly patients often present with multiple comorbidities necessitating numerous concurrent medications, which may elevate the risk of drug–drug interactions, consequently diminishing the potential benefits of CV prevention therapy (Figure 3) [35]. All primary prevention strategy decisions, and, in particular, the treatment of risk factors, should be considered, taking into account not only lone CV risks but also life expectancy and overall function, frailty, comorbidities, polypharmacy, and patient preferences [35,36]. However, considering the fact that these factors may be difficult to estimate, there are no strict criteria. In the case of a high risk, however, guidelines recommended the treatment of CVD risk factors [36].

Advocating for a healthy lifestyle is the optimal approach for elderly patients in primary prevention. Consistent engagement in physical activity has demonstrated considerable potential in mitigating and addressing adverse age-related impacts on the cCVr system. Additionally, certain pharmacological agents, including metformin, resveratrol, beta-blockers, and angiotensin-converting enzyme inhibitors, have emerged as potential anti-aging therapies with notable CV benefits [37]. These agents hold promise in ameliorating the age-related decline in CV function, thereby offering potential benefits in the management of CV health in the elderly population [28].

## 5. Cholesterol

Hypercholesterolemia is a causative factor for CV mortality at all ages; however, this association weakens after the age of 80 [38]. Nonetheless, an inverse correlation between all-cause mortality and LDL-C levels has been shown [39], although these data have not been confirmed by other analysis [39].

Statins stand as one of the foremost pharmacological interventions against atherosclerosis, often deployed as a first-line treatment. Substantial evidence supports their efficacy in reducing major vascular events among middle-aged populations, both with and without pre-existing ASCVD, where their usage is widespread. However, their utility in the primary prevention of CVD among healthy individuals aged 75 years and older remains a contentious topic. Existing evidence is sparse, offering limited support or excluding the benefits of this treatment in this particular demographic [40]. Indeed, older individuals are frequently under-represented in randomized controlled trials. A study focusing on 518 patients aged over 75 with type 2 diabetes mellitus, conducted as part of the Staged Diabetes Targeting Management Study, sheds light on this issue. Following a follow-up period of 6 years, the study revealed no significant correlation between statin use and all-cause mortality, even after meticulous adjustment for all conceivable confounding factors [41]. On the contrary, ASCVD stands as one of the foremost contributors to disability among older individuals. Consequently, CV prevention assumes paramount importance within this demographic to safeguard functional status and overall well-being [42].

However, statins seem to result in beneficial effects in the primary prevention of CVD in older populations [43,44,45], lowering the risk of coronary artery disease (CAD) but not all-cause or CV mortality or stroke [46].

In secondary prevention, a lower risk of CV events and mortality has been linked to the use of statins [47]. However, even in this setting, studies on very elderly populations are limited.

The PROSPER study, comprising patients aged 70–82 with ASCVD or high risk, treated with 40 mg of pravastatin or a placebo, demonstrated a noteworthy 15% reduction in the relative risk of ASCVD. Furthermore, subgroup analysis conducted in randomized trials has underscored a consistent reduction in relative risk across both young and elderly treated patients. In terms of secondary prevention, statins exhibit significant reductions in all-cause mortality, CV mortality, CAD, MI, and revascularization [46].

Contrasting evidence has also come from observational studies on statin treatment in the elderly [14,48,49,50,51,52,53,54,55,56,57,58,59,60,61] (Table 1).

Remarkably, the favorable correlation between statin use and the risk of all-cause mortality endure seven among individuals at advanced ages and remains consistent across genders, encompassing both men and women [62].

Individuals over 75 constitute a biologically diverse cohort, presenting unique challenges in clinical management. Due to the scarcity of evidence about this demographic, therapeutic decisions should be guided primarily by clinical judgment. Tailored medical care, including statins, should be carefully considered, taking into account individual characteristics and circumstances [63]. Factors such as frailty, susceptibility to side effects (especially muscle-related issues in the context of possible sarcopenia), cognitive impairment, variations in drug metabolism, underlying chronic comorbidities (including diabetes mellitus (DM)), potential pharmacological interactions resulting from polypharmacy, and patient preferences warrant particular attention. The therapy selection process should involve shared decision making wherein patient preferences and values are integral components [44,64]. Before initiating statin therapy, meticulous consideration should be given to selecting the appropriate type and dosage of statins [65].

Indeed, elderly patients are at an increased risk of experiencing side effects from statin treatment, due to the presence of multiple comorbidities and concurrent medications that may interact with statins. The complexity of their medical profile necessitates close monitoring and careful consideration of potential interactions to minimize the risk of adverse events, while maximizing the benefits of statin therapy [52]. The lowest effective dose should be used to achieve the lipid target [66]. According to European guidelines, LDL cholesterol values to be achieved in elderly patients are the same as those for adults [36], recommending initiating statin therapy, irrespective of age, when vascular disease coexists [36].

However, discontinuing statin treatment solely based on chronological age is unnecessary. Statin discontinuation is only advised within the framework of RCTs [64].

Like all preventive strategies, statin treatment should be ceased when palliative care is initiated [67].

Several large ongoing clinical trials will offer further insights into the potential advantages and drawbacks of initiating or discontinuing statins in older adults, using mortality, disability, and neurocognitive endpoints, along with standard CVD outcomes [44]. In the STAREE trial, patients aged more than 70 years without CVD, DM, or dementia randomly received atorvastatin (40 mg) or a placebo [68].

Regarding drugs different from statins, proprotein convertase subtilisin/kexin type 9 monoclonal antibodies (PCSK9 mAbs) have been shown to reduce LDL-C levels effectively. In a study on patients over 75 years receiving treatment for primary and secondary CV prevention, a reduction in LDL-C levels of 57% was observed in the elderly group, comparable to a 59% reduction in the control group within 6 months in the absence of significant adverse effects, showing efficacy and safety in elderly patients comparable to those in younger patients [69].

## 6. Diabetes Mellitus

Elderly diabetic individuals are at risk of developing the same complications as younger patients. Therapeutic goals (glycemic control and management of risk factors) will, therefore, be similar. However, this population is more likely to be frail, on polypharmacy, and with reduced functional abilities [70]. There is limited literature focusing on glycemic targets in elderly diabetic patients [71]. In the absence of randomized clinical trials, according to previous ESC guidelines, an elderly patient undergoing therapy with medications that do not pose a high risk of hypoglycemia should have a therapeutic goal of HbA1c < 7%; to achieve this target, fasting and pre-prandial glucose levels should be maintained within normal values without inducing hypoglycemia [72]. The goal should be more conservative (8–9%) in the presence of frailty, comorbidities, cognitive decline, limited life expectancy, multiple episodes of hypoglycemia, and complex pharmacological treatments [72]. HbA1c targets should be individualized based on the patient profile, taking into account comorbidities and age [72]. It would be advisable to avoid the use of medications that can induce the risk of hypoglycemia (sulfonylureas, repaglinide, insulin) in the elderly. Future research should focus on the risks of polypharmacy in elderly diabetic patients, particularly those who are frail and have comorbidities [72].

## 7. Arterial Hypertension

While blood pressure generally rises with age, uncertainty surrounding the value of treatment at advanced ages has historically hindered the management of AH. Concerns have centered on the higher potential for adverse events, particularly in very elderly individuals, often due to inadequate mechanisms for maintaining blood pressure and perfusion homeostasis. However, current treatments are generally better tolerated, even among frail patients with multiple comorbidities.

Recent trials, such as the Hypertension in the Very Elderly Trial (HYVET) and the Systolic Blood Pressure Intervention Trial (SPRINT), have demonstrated that aggressive blood pressure control can reduce CV mortality, disability, and overall mortality, even in elderly and very elderly patients [73,74].The data from the SPRINT have stirred some controversies due to variations in the characteristics of patients over 75 years compared to those encountered in real-world settings. Consequently, there is an imperative need for precise personalization of treatment goals, differentiating between “robust” and “frail and comorbid” elderly patients. Pharmacological treatment warrants careful monitoring, with a cautious approach to initiating combination therapy at the lowest-feasible dosage. In the case of very elderly patients, preference should be given to monotherapy, while avoiding medications such as loop diuretics and alpha-blockers due to the risk of orthostatic hypotension.

Determining the target blood pressure for treatment in elderly hypertensive patients is currently a clinically significant issue. Both the STEP study and SPRINT enrolled elderly participants but with some variations. Notably, previous stroke patients were excluded from both studies. However, it is worth noting that the STEP study enrolled subjects at a lower CV risk than SPRINT.

In the Chinese population aged 60–80 years with AH enrolled in the STEP study, intensive blood pressure management aimed at achieving a systolic blood pressure range of 110–130 mmHg decreased the primary composite CV endpoint and stroke occurrence. However, no evident impact was observed on CV mortality or adverse renal events. These findings differed from those of SPRINT, particularly in terms of the STEP population’s lower CV risk and prevalence of chronic kidney disease (CKD) [75].

According to previous European guidelines, the recommended target for systolic blood pressure in elderly individuals under treatment should typically be maintained between 130 and 139 mmHg, with the diastolic pressure ideally below 80 mmHg if well tolerated [59]. According to new 2023 ESH guidelines [76], in patients aged 18 to 79 years, the recommended office threshold for initiating drug treatment is a systolic blood pressure (SBP) of 140 mmHg and/or a diastolic blood pressure (DBP) of 90 mmHg (IA). For patients over 80 years, the SBP threshold is set at 160 mmHg (IB). However, in patients aged 80 years and older, a lower SBP threshold, in the range of 140–159 mmHg, may be considered (IIC).

For patients aged 65 to 79 years, the primary treatment goal is to lower blood pressure to below 140/80 mmHg (IA). Nonetheless, lowering blood pressure to below 130/80 mmHg can be contemplated if treatment is well tolerated (IIB). In patients aged 65 to 79 years with isolated systolic hypertension, the primary objective is to lower the SBP to a range of 140–150 mmHg (IA). However, a reduction in the office SBP to the range of 130–139 mmHg should be considered cautiously if well tolerated, particularly if the DBP is already below 70 mmHg (IB).

For patients aged 80 years and older, the SBP should be lowered to a range of 140–150 mmHg (IA). Similar to the younger age group, a reduction in the office SBP to the range of 130–139 mmHg may be considered if well tolerated, albeit cautiously if the DBP is already below 70 mmHg (IIB) [76].

## 8. Antiplatelet Therapy

Various physiological aging-related pathophysiological mechanisms have been identified as contributing factors to the heightened thromboembolic risk in the elderly. These mechanisms encompass oxidative stress, endothelial dysfunction, activation of platelets, and the coagulation cascade, as well as diminished responsiveness to pharmacological interventions [77]. There remains a lack of consensus regarding the most appropriate antithrombotic treatment for the elderly, particularly considering the challenges associated with managing bleeding risks in this high-risk subgroup of patients.

The daily use of low-dose aspirin does not significantly prevent disability or CVD among adults over 70 years old but significantly increases bleeding risk [78,79,80].

Therefore, antiplatelet treatment should not be routinely used for CV primary prevention [77]. On the contrary, aspirin use for CV primary prevention in older adults should be individualized, taking into account the patient’s profile [81,82].

Different data come from secondary prevention. The incidence of acute coronary syndrome (ACS) is also shifting toward older age [83]. In the EYESHOT study, the elderly represented about one-third of the patients hospitalized for ACS in Italian cardiology departments [84]. Greater antiplatelet activity of ticagrelor was demonstrated in the elderly compared to that of clopidogrel, resulting in less platelet hyperreactivity [85].

Newer P2Y12 inhibitors used in elderly patients should be evaluated carefully by comparing ischemia and bleeding and assessing frailty [86]. In elderly patients with acute coronary syndrome (ACS), low-dose prasugrel or clopidogrel, shorter dual-antiplatelet therapy, and avoiding pretreatment before stent placement should be considered. However, elderly patients have been shown to have less likelihood of undergoing coronary angiography and angioplasty despite their higher mortality. The EYESHOT study recognizes aging for more than 75 years as a new P2Y12 inhibitor under-prescription determinant. For those over 80 with non-ST-segment elevation myocardial infarction (NSTEMI), invasive treatment is more effective than conservative approaches, with similar bleeding rates [83].

## 9. Anticoagulant Therapy

The prevalence of AF increases with age, representing a significant challenge in the elderly [87]. Oral anticoagulants (OACs) are used in patients with AF, pulmonary embolism, and venous thromboembolism (VTE). Notably, the elderly have been shown to benefit from OACs [88].

According to the BAFTA study, patients over 75 treated with warfarin have a better outcome than those treated with aspirin [89]. In patients on vitamin-K-dependent anticoagulants (VKAs), factors such as comorbidities, hematological disorders, malnutrition, and polypharmacy have been reported to influence INR variability [90]. Direct oral anticoagulants (DOACs) resulted in non-inferior or superior efficacy of warfarin, with a reduced risk of major bleeding and a better overall risk–benefit profile, and are increasingly prescribed in the elderly [91]. In patients over 75 years old from a French registry, DOAC users were likely to be men, more with chronic diseases and drugs per prescription [92,93].

However, despite their proven efficacy, these medications are not sufficiently used among elderly patients [88]. Safe and effective anticoagulation approaches are often avoided in individuals at the greatest risk of embolic strokes, for fear of complications [94]. A tailored AF approach should be adopted in elderly patients, considering the risk of complications and evaluating both OACs and a left atrial appendage occlusion strategy [94]. Moreover, hemorrhagic risk should be carefully evaluated in the elderly [77].

## 10. Electrical Devices

In recent years, there has been an exponential increase in patients undergoing cardiac electronic device implantation [95]. Approximately 75% of pacemakers (PMs) and 30–35% of implantable cardioverter-defibrillators (ICDs) are implanted in individuals over 75 years old [96]. However, the data supporting the clinical effectiveness of these devices in this age group, which is poorly represented in large trials, are contradictory.

Aging processes, such as apoptosis and fibroadipose infiltration of the sinoatrial node, along with polypharmacy, severely depress automaticity and nodal conduction. The prevalence of symptomatic sinus bradycardia is 1/600 after 65 years, with a 70–80% pacemaker implantation rate for sinoatrial dysfunction. Normal aging causes calcifications in the atrioventricular node, the His bundle, and branches. With age, there is an increased incidence of PR interval prolongation, right and left bundle branch blocks (BBBs), and atrioventricular (AV) blocks. In the elderly, there is a progressive increase in ventricular arrhythmias regardless of the presence of underlying heart disease [96]. The most common cause of sustained ventricular arrhythmias in the elderly is CAD, followed by dilated cardiomyopathy. Sudden cardiac death (SCD) in the elderly is ischemic in 88% of cases, and age is an unfavorable prognostic factor (survival rate < 5%), often associated with electromechanical dissociation and asystole. ICDs represent a widespread strategy to prevent SCD in high-risk patients. About 80% of patients eligible for ICDs are octogenarians. According to a meta-analysis of RCTs [97], in which the percentage of those over 75 varied from 9 to 17%, ICD implantation reduces all-cause mortality in patients ≥75 years old. Recently, one of the largest real-life registries on defibrillators in primary prevention has shown that sudden cardiac death and the delivery of appropriate therapies are similar across different age groups [98]. However, non-arrhythmic mortality is a confounding factor. Indeed, since in many studies, the survival of the elderly is <5 years, the actual effectiveness of ICDs in increasing life expectancy seems marginal. The benefits of the defibrillator in the primary prevention of SCDs are apparent only in medium- to long-term follow-up (2–5 years) [99,100]. Additionally, a meta-analysis [94] did not show a significant survival benefit in elderly patients undergoing defibrillator implantation in primary prevention. Regarding safety, in a study of patients undergoing primary prevention defibrillator implantation, the incidence of adverse events and in-hospital mortality increased from 2.8% below 65 years to 4.5% after 80 years; however, most complications were minor [101].

For individuals aged 80 and above, the short-term mortality rates at 1 year and 2 years post-implantation of ICDs were recorded at 56% and 72%, respectively [102].

In a retrospective analysis involving 145 elderly patients aged 72 years and older who underwent ICD implantation, it was determined that a diminished baseline ejection fraction (EF) emerges as a significant independent predictor of mortality [103]. In a multicenter study conducted in France to evaluate patients who received an ICD for primary prevention [104], a comparable proportion of patients in the elderly group and controls received at least one appropriate therapy. There was a tendency toward more early perioperative events among the elderly, yet no significant increase in late complications was observed. Additionally, a risk score based on 10 variables was developed to stratify the risk of peri-procedural complications accurately [105].

Although guidelines consider the implantation of defibrillators in the elderly “rarely appropriate”, no specific age cutoff for the primary prevention implantable cardioverter-defibrillator is specified, indicating that the sole criterion for selection is an expected life expectancy of more than 1year [106]. A careful multidisciplinary evaluation is needed to guide patient selection for ICD implantation in the elderly population [104]. ICD intervention in the elderly remains debatable when severe comorbidities occur [102]. In elderly patients who are expected to not have benefits due to age and comorbidities, ICD implantation for primary prevention may be avoided [107].

## 11. Vaccination

Preventing severe complications arising from influenza is imperative, particularly within the elderly demographic, where advancing age escalates the susceptibility to mortality stemming from respiratory illnesses associated with influenza. An examination encompassing 5608 Japanese patients aged ≥65 years diagnosed with influenza underscored a significant association between influenza vaccination and a reduction in adverse composite outcomes. This relationship was notably pronounced among individuals aged 80 years and older, as well as those afflicted with CVD [108].

## 12. Cardiac Rehabilitation in the Elderly

Comprehensive cardiac rehabilitation programs encompass multifactorial components, such as exercise and nutritional interventions, aimed at optimizing CV risk reduction, promoting healthy behaviors and an active lifestyle, reducing disability, and enhancing health and well-being. Increased survival rates following ACS and innovations in both catheter-based and surgical interventions have resulted in a higher number of frail individuals requiring cardiac rehabilitation [109].

New preconditioning rehabilitation models have been suggested for frail elderly patients prior to surgery to improve functional outcomes and reduce hospital stays. Individual tailoring of cardiac rehabilitation is fundamental to providing high-quality care for the elderly.

Despite the strong recommendations for cardiac rehabilitation, participation rates across Europe remain low, particularly among the elderly [110].

## 13. Real-World Data

Data from the National Health and Nutrition Examination Survey on 1566 elderly aged ≥75 years aimed to investigate secondary CAD prevention and drug adherence [47] reported the achievement of target benchmarks for blood pressure, low-density lipoprotein cholesterol, smoking cessation, adherence to recommended alcohol intake, and maintaining glycated hemoglobin levels in most patients. Nevertheless, achievement rates were notably lower for the body mass index, waist circumference, and adequate physical activity. Additionally, the use of prescribed therapy for BB, angiotensin-converting enzyme inhibitors/angiotensin receptor blockers, statins, and antiplatelet drugs was observed in 54.41%, 49.36%, 54.79%, and 19.03% of cases, respectively, with merely 6.26% receiving all four medications. Consequently, a substantial proportion of elderly CAD patients demonstrated inadequate overall control of crucial CAD risk factors [47].

In a study involving 424 randomly selected individuals aged 60 years and older, a questionnaire-based survey was conducted to assess self-efficacy rates, lifestyle practices, and respondents’ knowledge of CV risk factors [111]. Physical exercise, smoking, soft drink consumption, and alcohol use were reported in 1.7%, 4.5%, 18.2%, and 13.2% of the population, respectively. Excess salt use and eating outside were reported in 92.7% and 64.6% of the cases. Overall, 58.5%, 30.0%, and 11.6% of the respondents had fair, good, and poor lifestyle practices [111]. In a prospective 10-year study on 418 elderly patients living in Central Brazil, AH occurred in most people, accounting for 81.6% [112]. Among the participants, 44% engaged in irregular physical activity, and 43.3% reported smoking. Following a mean follow-up period of 8.38 years (±2.82), age, hypertension, and smoking were identified as risk factors associated with decreased survival, while physical activity emerged as a protective factor [112].

## 14. Conclusions

The great advances in medicine have led to a spectacular increase in life expectancy, posing significant problems in terms of the appropriateness and sustainability of services for Western societies. The scenario of industrialized societies is characterized, at the dawn of the third millennium, by the progressive aging of the population; the spread of degenerative diseases, particularly CV ones; and a technological explosion that influences clinical practice. Debate exists regarding the uniform significance of CV risk factors across different age groups, questioning the necessity of treatment and whether a threshold age exists beyond which treatment may no longer be deemed worthwhile [113]. In older adults, advancing age is the predominant CV risk factor. Moreover, there are no disparities in the distribution of CV risk factors between older and younger individuals. Targeted prevention interventions are ideally initiated at a younger age; however, they are also beneficial in older adults, contributing to prolonging life and enhancing the quality of life [114]. Various pharmacological treatments have been demonstrated to reduce CV events in elderly individuals substantially [38,43,71,72,73,115,116].

In terms of prevention, there is no justification for inertia in the approach to elderly patients. However, the literature on randomized controlled trials involving the elderly population is notably limited. Many analyses have primarily focused on subgroups of patients included in controlled studies, which should ideally only serve as hypothesis generating. Furthermore, trials specifically recruiting elderly patients often involve individuals who are considerably frailer than the general elderly population, complicating the generalization of findings to the broader demographic. As such, the role of prevention, particularly primary prevention, in the elderly warrants a more thorough evaluation through the design of tailored study protocols for this demographic. Whether primary/secondary prevention of CV risk factors may fit in the elderly population’s personalized treatment or would influence their quality of life are important issues in geriatrics that should also be investigated.

In conclusion, the growing number and proportion of elderly individuals in the population represent both an accomplishment and a challenge to the sustainability of current welfare systems, known as the “longevity shock”. Urgent measures are needed to halt the ongoing defunding of healthcare and to assign the management of national health services to highly competent professionals capable of strategic planning for systematic interventions in socio-health policies. These interventions should prioritize research, healthcare provision, and the overall well-being of the elderly population. Emphasis should be placed on avoiding hospitalizations by favoring community-based interventions focused on prevention, rehabilitation, and environmental adaptations and providing economic, social, and motivational support for elderly individuals and their families. A comprehensive multidimensional assessment appears to be the most effective approach currently available for devising tailored treatment plans based on the baseline condition and comorbidities of each elderly patient [117].

## Figures and Tables

**Table 1:** Statin treatment in the elderly.

Authors	Year	Patients	Median Age (Years)	Median Follow-Up (Years)	Main Results
Bezin et al. [48]	2019	7284 new users of statins	≥75	4.7	Statin use: -↓Risk of outcomes in the primary-prevention-with-modifiable-risk-factors group (*p* < 0.01) and in the secondary prevention group (*p* < 0.01) -Not in the primary-prevention-without-modifiable-risk-factors group (*p* = 0.92)
Zhou et al. [49]	2020	18,096 healthy adults	≥70	4.7	Statin use: -↓Risk of physical disability and CV outcomes
Giral et al. [61]	2019	120,173 in primary prevention	≥75	2.4	Statin discontinuation: -↑ 33% Risk of CV hospitalizations
Jun et al. [60,61]	2019	11,017 with CVD; 55,085 controls	≥75		Statin use: -↓Composite outcome -↓Risk of stroke -↓Risk of all-cause death -No effect on CVD or all-cause death
Kim et al. [59]	2020	1370	78		Statin use >5 years: -↓Risk of all-cause mortality (*p* = 0.01) -No major adverse cardiovascular events (*p* = 0.36)
Kim et al. [58]	2020	Elderly Koreans	≥75		-↓ CV events -↓ All-cause mortality rate
Cho et al. [57]	2021	81,729 without CVD	≥75		-↓ Risk of CV death (*p* < 0.001)
Gitsels et al. [56]	2021	General population	≥60		Statin use: -↓ All-cause mortality
Lavie et al. [55]	2021	42,767 new users of statins	14% ≥70		Statin use: -↓CV events and mortality for the highest-adherence group
Sarraju et al. [54]	2021	54,066 without ASCVD	65–79		- Inferior use of statins in patients >75 years old compared to younger ones
		10,288 without ASCVD	≥70		
Bergami et al. [14]	2022	5619 without CVD	≥65		Statin use: -↓STEMI risk
Funaki et al. [53]	2023	Patients without CVD	≥65		Statin use: -↓Risk of all-cause mortality
Horikoshi et al. [52]	2022	1676 patients with CAD who underwent PCI	≥75		Statin treatment: -↓ MACE occurrence (*p* < 0.001)
Kim et al. [51]	2022	1370	≥75		Statin use: -↓ Mortality and MACE
Lee et al. [50]	2022	Patients without CVD	≥65		Statin use: -↓ Risk MI and stroke and mortality

## References

[1] Murray C.J., Barber R.M., Foreman K.J., Abbasoglu Ozgoren A., Abd-Allah F., Abera S.F., Aboyans V., Abraham J.P., Abubakar I., Abu-Raddad L.J. (2015). Global, regional, and national disability-adjusted life years (DALYs) for 306 diseases and injuries and healthy life expectancy (HALE) for 188 countries, 1990–2013: Quantifying the epidemiological transition. Lancet.

[2] Li X., Man J., Chen H., Yang X. (2022). Spatiotemporal trends of disease burden of edentulism from 1990 to 2019: A global, regional, and national analysis. Front Public Health.

[3] Ben-Shlomo Y., Cooper R., Kuh D. (2016). The last two decades of life course epidemiology, and its relevance for research on ageing. Int. J. Epidemiol..

[4] Lucà F., Pavan D., Gulizia M.M., Manes M.T., Abrignani M.G., Benedetto F.A., Bisceglia I., Brigido S., Caldarola P., Calvanese R. (2024). Gender discrepancy: Time to implement gender-based clinical management. G. Ital. Cardiol..

[5] Lucà F., Abrignani M.G., Parrini I., Di Fusco S.A., Giubilato S., Rao C.M., Piccioni L., Cipolletta L., Passaretti B., Giallauria F. (2022). Update on Management of Cardiovascular Diseases in Women. J. Clin. Med..

[6] Yadegarfar M.E., Jagger C., Duncan R., Fouweather T., Hanratty B., Parker S., Robinson L. (2018). Use of primary care and other healthcare services between age 85 and 90 years: Longitudinal analysis of a single-year birth cohort, the Newcastle 85+ study. BMJ Open.

[7] Tampubolon G. (2015). Delineating the third age: Joint models of older people’s quality of life and attrition in Britain 2002–2010. Aging Ment Health.

[8] Nakatani H. (2023). Aging and Health: Aiming at Healthy Longevity. Global Health Essentials.

[9] Arbeev K.G., Akushevich I., Kulminski A.M., Ukraintseva S.V., Yashin A.I. (2014). Joint Analyses of Longitudinal and Time-to-Event Data in Research on Aging: Implications for Predicting Health and Survival. Front. Public Health.

[10] Cannata A., Merlo M., Artico J., Gentile P., Camparini L., Cristallini J., Porcari A., Loffredo F., Sinagra G. (2018). Cardiovascular aging: The unveiled enigma from bench to bedside. J. Cardiovasc. Med..

[11] Rosengren A. (2012). Better treatment and improved prognosis in elderly patients with AMI: But do registers tell the whole truth?. Eur. Heart J..

[12] Abrignani M.G., Aiello A., Colivicchi F., Lucà F., Fattirolli F., Gulizia M.M., Nardi F., Pino P.G., Gregorio G. (2020). Cardiovascular prevention in the elderly: Limitations and opportunities. G. Ital. Cardiol..

[13] Nakamura E., Miyao K. (2007). A method for identifying biomarkers of aging and constructing an index of biological age in humans. J. Gerontol. A Biol. Sci. Med. Sci..

[14] Bergami M., Cenko E., Yoon J., Mendieta G., Kedev S., Zdravkovic M., Vasiljevic Z., Miličić D., Manfrini O., van der Schaar M. (2022). Statins for primary prevention among elderly men and women. Cardiovasc. Res..

[15] Bonanad C., Fernández-Olmo R., García-Blas S., Alarcon J.A., Díez-Villanueva P., Mansilla C.R., García-Pardo H., Toledo P., Ayesta A., Pereira E. (2022). Cardiovascular prevention in elderly patients. J. Geriatr. Cardiol..

[16] Rich M.W., Chyun D.A., Skolnick A.H., Alexander K.P., Forman D.E., Kitzman D.W., Maurer M.S., McClurken J.B., Resnick B.M., Shen W.K. (2016). Knowledge Gaps in Cardiovascular Care of the Older Adult Population: A Scientific Statement from the American Heart Association, American College of Cardiology, and American Geriatrics Society. Circulation.

[17] Forman D.E., Arena R., Boxer R., Dolansky M.A., Eng J.J., Fleg J.L., Haykowsky M., Jahangir A., Kaminsky L.A., Kitzman D.W. (2017). Prioritizing Functional Capacity as a Principal End Point for Therapies Oriented to Older Adults with Cardiovascular Disease: A Scientific Statement for Healthcare Professionals From the American Heart Association. Circulation.

[18] Fried L.P., Tangen C.M., Walston J., Newman A.B., Hirsch C., Gottdiener J., Seeman T., Tracy R., Kop W.J., Burke G. (2001). Frailty in older adults: Evidence for a phenotype. J. Gerontol. A Biol. Sci. Med. Sci..

[19] Rockwood K., Wolfson C., McDowell I. (2001). The Canadian Study of Health and Aging: Organizational lessons from a national, multicenter, epidemiologic study. Int. Psychogeriatr..

[20] Nguyen T.N., Ahmad F., Lindley R.I. (2024). Frailty in clinical drug trials: Frailty assessments, subgroup analyses and outcomes. Br. J. Clin. Pharmacol..

[21] Adachi T., Tsunekawa Y., Tanimura D. (2022). Association among mild cognitive impairment, social frailty, and clinical events in elderly patients with cardiovascular disease. Heart Lung.

[22] Ruiz M., Bottle A., Long S., Aylin P. (2015). Multi-Morbidity in Hospitalised Older Patients: Who Are the Complex Elderly?. PLoS ONE.

[23] Rozzini R., Frisoni G.B., Ferrucci L., Barbisoni P., Sabatini T., Ranieri P., Guralnik J.M., Trabucchi M. (2002). Geriatric Index of Comorbidity: Validation and comparison with other measures of comorbidity. Age Ageing.

[24] Guralnik J.M., Simonsick E.M., Ferrucci L., Glynn R.J., Berkman L.F., Blazer D.G., Scherr P.A., Wallace R.B. (1994). A short physical performance battery assessing lower extremity function: Association with self-reported disability and prediction of mortality and nursing home admission. J. Gerontol..

[25] Borson S., Scanlan J., Brush M., Vitaliano P., Dokmak A. (2000). The mini-cog: A cognitive ‘vital signs’ measure for dementia screening in multi-lingual elderly. Int. J. Geriatr. Psychiatry.

[26] American Geriatrics Society Expert Panel on the Care of Older Adults with Multimorbidity (2012). Patient-centered care for older adults with multiple chronic conditions: A stepwise approach from the American Geriatrics Society: American Geriatrics Society Expert Panel on the Care of Older Adults with Multimorbidity. J. Am. Geriatr. Soc..

[27] Yan M., Sun S., Xu K., Huang X., Dou L., Pang J., Tang W., Shen T., Li J. (2021). Cardiac Aging: From Basic Research to Therapeutics. Oxid. Med. Cell. Longev..

[28] Howlett L.A., Jones S.A., Lancaster M.K. (2022). Pharmacy and Exercise as Complimentary Partners for Successful Cardiovascular Ageing. Curr. Vasc. Pharmacol..

[29] Faggiano P., Antonini-Canterin F., Erlicher A., Romeo C., Cervesato E., Pavan D., Piazza R., Huang G., Nicolosi G.L. (2003). Progression of aortic valve sclerosis to aortic stenosis. Am. J. Cardiol..

[30] Barasch E., Gottdiener J.S., Larsen E.K., Chaves P.H., Newman A.B., Manolio T.A. (2006). Clinical significance of calcification of the fibrous skeleton of the heart and aortosclerosis in community dwelling elderly. The Cardiovascular Health Study (CHS). Am. Heart J..

[31] Kohsaka S., Jin Z., Rundek T., Boden-Albala B., Homma S., Sacco R.L., Di Tullio M.R. (2008). Impact of mitral annular calcification on cardiovascular events in a multiethnic community: The Northern Manhattan Study. JACC Cardiovasc. Imaging.

[32] Bursi F., Weston S.A., Redfield M.M., Jacobsen S.J., Pakhomov S., Nkomo V.T., Meverden R.A., Roger V.L. (2006). Systolic and diastolic heart failure in the community. JAMA.

[33] Lucà F., Oliva F., Abrignani M.G., Di Fusco S.A., Gori M., Giubilato S., Ceravolo R., Temporelli P.L., Cornara S., Rao C.M. (2024). Heart Failure with Preserved Ejection Fraction: How to Deal with This Chameleon. J. Clin. Med..

[34] Mehta S., Jackson R., Poppe K., Kerr A.J., Pylypchuk R., Wells S. (2020). How do cardiovascular risk prediction equations developed among 30-74 year olds perform in older age groups? A validation study in 125,000 people aged 75–89 years. J. Epidemiol. Community Health.

[35] Palmiero P., Zito A., Maiello M., Cecere A., Mattioli A.V., Pedrinelli R., Scicchitano P., Ciccone M.M. (2019). Primary Prevention of Cardiovascular Risk in Octogenarians by Risk Factors Control. Curr. Hypertens. Rev..

[36] Visseren F.L.J., Mach F., Smulders Y.M., Carballo D., Koskinas K.C., Bäck M., Benetos A., Biffi A., Boavida J.M., Capodanno D. (2021). 2021 ESC Guidelines on cardiovascular disease prevention in clinical practice. Eur. Heart J..

[37] Parrini I., Lucà F., Rao C.M., Cacciatore S., Riccio C., Grimaldi M., Gulizia M.M., Oliva F., Andreotti F. (2024). How to Manage Beta-Blockade in Older Heart Failure Patients: A Scoping Review. J. Clin. Med..

[38] Lewington S., Whitlock G., Clarke R., Sherliker P., Emberson J., Halsey J., Qizilbash N., Peto R., Collins R. (2007). Blood cholesterol and vascular mortality by age, sex, and blood pressure: A meta-analysis of individual data from 61 prospective studies with 55,000 vascular deaths. Lancet.

[39] Ravnskov U., Diamond D.M., Hama R., Hamazaki T., Hammarskjöld B., Hynes N., Kendrick M., Langsjoen P.H., Malhotra A., Mascitelli L. (2016). Lack of an association or an inverse association between low-density-lipoprotein cholesterol and mortality in the elderly: A systematic review. BMJ Open.

[40] Lucchi T. (2021). Dyslipidemia and prevention of atherosclerotic cardiovascular disease in the elderly. Minerva Med..

[41] Fan Y., Wang J., Wu H., Dai L., Wang L., Gu L. (2023). Effect of statin treatment on mortality in elderly patients with type 2 diabetes mellitus patients: A retrospective cohort study. BMC Geriatr..

[42] Cobos-Palacios L., Sanz-Cánovas J., Muñoz-Ubeda M., Lopez-Carmona M.D., Perez-Belmonte L.M., Lopez-Sampalo A., Gomez-Huelgas R., Bernal-Lopez M.R. (2021). Statin Therapy in Very Old Patients: Lights and Shadows. Front. Cardiovasc. Med..

[43] Reiner Z. (2014). Primary prevention of cardiovascular disease with statins in the elderly. Curr. Atheroscler. Rep..

[44] Wierzbicki A.S. (2024). Preventive cardiology for the aging population: How can we better design clinical trials of statins?. Expert Rev. Cardiovasc. Ther..

[45] Savarese G., Gotto A.M., Paolillo S., D’Amore C., Losco T., Musella F., Scala O., Marciano C., Ruggiero D., Marsico F. (2013). Benefits of statins in elderly subjects without established cardiovascular disease: A meta-analysis. J. Am. Coll. Cardiol..

[46] Ponce O.J., Larrea-Mantilla L., Hemmingsen B., Serrano V., Rodriguez-Gutierrez R., Spencer-Bonilla G., Alvarez-Villalobos N., Benkhadra K., Haddad A., Gionfriddo M.R. (2019). Lipid-Lowering Agents in Older Individuals: A Systematic Review and Meta-Analysis of Randomized Clinical Trials. J. Clin. Endocrinol. Metab..

[47] Zhai C., Hou K., Li R., Hu Y., Zhang J., Zhang Y., Wang L., Zhang R., Cong H. (2020). Efficacy of statin treatment based on cardiovascular outcomes in elderly patients: A standard meta-analysis and Bayesian network analysis. J. Int. Med. Res..

[48] Bezin J., Moore N., Mansiaux Y., Steg P.G., Pariente A. (2019). Real-Life Benefits of Statins for Cardiovascular Prevention in Elderly Subjects: A Population-Based Cohort Study. Am. J. Med..

[49] Zhou Z., Ofori-Asenso R., Curtis A.J., Breslin M., Wolfe R., McNeil J.J., Murray A.M., Ernst M.E., Reid C.M., Lockery J.E. (2020). Association of Statin Use with Disability-Free Survival and Cardiovascular Disease Among Healthy Older Adults. J. Am. Coll. Cardiol..

[50] Lee Y.B., Koo M., Noh E., Hwang S.Y., Kim J.A., Roh E., Hong S.H., Choi K.M., Baik S.H., Cho G.J. (2022). Myocardial Infarction, Stroke, and All-Cause Mortality according to Low-Density Lipoprotein Cholesterol Level in the Elderly, a Nationwide Study. Diabetes Metab. J..

[51] Kim S., Choi H., Won C.W. (2022). Moderate-intensity statin use for primary prevention for more than 5 years is associated with decreased all-cause mortality in 75 years and older. Arch. Gerontol. Geriatr..

[52] Horikoshi T., Nakamura T., Yoshizaki T., Nakamura J., Watanabe Y., Uematsu M., Makino A., Kobayashi T., Saito Y., Obata J.E. (2023). A Stratified Analysis of the Risk Associated with Low Body Mass Index for Patients After Percutaneous Coronary Intervention. J. Atheroscler. Thromb..

[53] Funaki D., Kaneda H., Miyakoshi A., Saito K., Sasaki H., Nakatani E. (2023). Identification of subgroups within a Japanese older adult population for whom statin therapy is effective in reducing mortality. PLoS ONE.

[54] Sarraju A., Spencer-Bonilla G., Chung S., Gomez S., Li J., Heidenreich P., Palaniappan L., Rodriguez F. (2022). Statin Use in Older Adults for Primary Cardiovascular Disease Prevention Across a Spectrum of Cardiovascular Risk. J. Gen. Intern. Med..

[55] Lavie G., Hoshen M., Leibowitz M., Benis A., Akriv A., Balicer R., Reges O. (2021). Statin Therapy for Primary Prevention in the Elderly and Its Association with New-Onset Diabetes, Cardiovascular Events, and All-Cause Mortality. Am. J. Med..

[56] Gitsels L.A., Bakbergenuly I., Steel N., Kulinskaya E. (2021). Do statins reduce mortality in older people? Findings from a longitudinal study using primary care records. Fam. Med. Community Health.

[57] Cho Y., Jeong Y., Seo D.H., Ahn S.H., Hong S., Suh Y.J., Kim S.H. (2021). Use of statin for the primary prevention of cardiovascular outcomes in elderly patients: A propensity-matched cohort study. Atherosclerosis.

[58] Kim K., Lee S.H. (2020). Effects of Statins for Primary Prevention in the Elderly: Recent Evidence. J. Lipid Atheroscler..

[59] Kim S., Choi H., Won C.W. (2020). Effects of Statin Use for Primary Prevention among Adults Aged 75 Years and Older in the National Health Insurance Service Senior Cohort (2002–2015). Ann. Geriatr. Med. Res..

[60] Jun J.E., Cho I.J., Han K., Jeong I.K., Ahn K.J., Chung H.Y., Hwang Y.C. (2019). Statins for primary prevention in adults aged 75 years and older: A nationwide population-based case-control study. Atherosclerosis.

[61] Giral P., Neumann A., Weill A., Coste J. (2019). Cardiovascular effect of discontinuing statins for primary prevention at the age of 75 years: A nationwide population-based cohort study in France. Eur. Heart J..

[62] Awad K., Mohammed M., Zaki M.M., Abushouk A.I., Lip G.Y.H., Blaha M.J., Lavie C.J., Toth P.P., Jukema J.W., Sattar N. (2021). Association of statin use in older people primary prevention group with risk of cardiovascular events and mortality: A systematic review and meta-analysis of observational studies. BMC Med..

[63] Miura S.I., Katsuda Y., Sugihara M., Ike A., Nishikawa H., Kawamura A. (2020). A Strict Target for Low-Density Lipoprotein Cholesterol May not Be Necessary for Secondary Prevention of Cardiovascular Disease in All Elderly Patients With Dyslipidemia. Cardiol. Res..

[64] Bétrisey S., Baretella O., Blum M., Aubert C.E., Rodondi N. (2022). Should dyslipidemia be treated in the elderly and very old people?. Rev. Med. Suisse.

[65] Fiore V., Barucca A., Barraco S., Triggiani D., Tragni D., Piazzolla G., Triggiani V., Carbotta G., Lisco G. (2023). Dyslipidemia and Cardiovascular Prevention in the Elderly: A Balance between Benefits and Risks of Statin Treatment in a Specific Population. Endocr. Metab. Immune Disord. Drug Targets.

[66] Lucà F., Oliva F., Rao C.M., Abrignani M.G., Amico A.F., Di Fusco S.A., Caretta G., Di Matteo I., Di Nora C., Pilleri A. (2023). Appropriateness of Dyslipidemia Management Strategies in Post-Acute Coronary Syndrome: A 2023 Update. Metabolites.

[67] Strandberg T.E. (2019). Role of Statin Therapy in Primary Prevention of Cardiovascular Disease in Elderly Patients. Curr. Atheroscler. Rep..

[68] Zoungas S., Curtis A., Spark S., Wolfe R., McNeil J.J., Beilin L., Chong T.T., Cloud G., Hopper I., Kost A. (2023). Statins for extension of disability-free survival and primary prevention of cardiovascular events among older people: Protocol for a randomised controlled trial in primary care (STAREE trial). BMJ Open.

[69] Giladi E., Israel R., Daud W., Gurevitz C., Atamna A., Pereg D., Assali A., Elis A. (2024). Anti PCSK9 Monoclonal Antibody Treatment in Elderly Patients: A Real-world Clinical Experience. Isr. Med. Assoc. J..

[70] Kirkman M.S., Briscoe V.J., Clark N., Florez H., Haas L.B., Halter J.B., Huang E.S., Korytkowski M.T., Munshi M.N., Odegard P.S. (2012). Diabetes in older adults: A consensus report. J. Am. Geriatr. Soc..

[71] Lipska K.J., Krumholz H., Soones T., Lee S.J. (2016). Polypharmacy in the Aging Patient: A Review of Glycemic Control in Older Adults with Type 2 Diabetes. JAMA.

[72] Cosentino F., Grant P.J., Aboyans V., Bailey C.J., Ceriello A., Delgado V., Federici M., Filippatos G., Grobbee D.E., Hansen T.B. (2020). 2019 ESC Guidelines on diabetes, pre-diabetes, and cardiovascular diseases developed in collaboration with the EASD. Eur. Heart J..

[73] Williamson J.D., Supiano M.A., Applegate W.B., Berlowitz D.R., Campbell R.C., Chertow G.M., Fine L.J., Haley W.E., Hawfield A.T., Ix J.H. (2016). Intensive vs Standard Blood Pressure Control and Cardiovascular Disease Outcomes in Adults Aged ≥75 Years: A Randomized Clinical Trial. JAMA.

[74] Hazra N.C., Rudisill C., Jackson S.H., Gulliford M.C. (2019). Cost-Effectiveness of Antihypertensive Therapy in Patients Older Than 80 Years: Cohort Study and Markov Model. Value Health.

[75] Modesti P.A. (2021). What we learned from STEP that we didn’t already know from SPRINT. Int. J. Cardiol. Cardiovasc. Risk Prev..

[76] Mancia G., Kreutz R., Brunström M., Burnier M., Grassi G., Januszewicz A., Muiesan M.L., Tsioufis K., Agabiti-Rosei E., Algharably E.A.E. (2023). 2023 ESH Guidelines for the management of arterial hypertension The Task Force for the management of arterial hypertension of the European Society of Hypertension: Endorsed by the International Society of Hypertension (ISH) and the European Renal Association (ERA). J. Hypertens..

[77] Valeriani E., Bartimoccia S., Pignatelli P., Pastori D. (2023). Aging and Antithrombotic Treatment. Antioxid. Redox Signal..

[78] McNeil J.J., Wolfe R., Woods R.L., Tonkin A.M., Donnan G.A., Nelson M.R., Reid C.M., Lockery J.E., Kirpach B., Storey E. (2018). Effect of Aspirin on Cardiovascular Events and Bleeding in the Healthy Elderly. N. Engl. J. Med..

[79] Patel N.J., Baliga R.R. (2020). Role of Aspirin for Primary Prevention in Persons with Diabetes Mellitus and in the Elderly. Curr. Cardiol. Rep..

[80] O’Sullivan J.W. (2019). Aspirin for the primary prevention of cardiovascular disease in the elderly. BMJ Evid. Based Med..

[81] Darraj A. (2024). Effect of Low-Dose Aspirin on the Elderly. Cureus.

[82] Lewis J., Bethishou L., Tsu L.V. (2019). Aspirin Use for Primary Prevention of Cardiovascular Disease in Older Patients: A Review of Clinical Guidelines and Updated Evidence. Sr. Care Pharm..

[83] Montilla Padilla I., Martín-Asenjo R., Bueno H. (2017). Management of Acute Coronary Syndromes in Geriatric Patients. Heart Lung Circ..

[84] De Luca L., Leonardi S., Cavallini C., Lucci D., Musumeci G., Caporale R., Abrignani M.G., Lupi A., Rakar S., Gulizia M.M. (2015). Contemporary antithrombotic strategies in patients with acute coronary syndrome admitted to cardiac care units in Italy: The EYESHOT Study. Eur. Heart J. Acute Cardiovasc. Care.

[85] Verdoia M., Pergolini P., Rolla R., Nardin M., Schaffer A., Barbieri L., Marino P., Bellomo G., Suryapranata H., De Luca G. (2016). Advanced age and high-residual platelet reactivity in patients receiving dual antiplatelet therapy with clopidogrel or ticagrelor. J. Thromb. Haemost..

[86] Capodanno D., Angiolillo D.J., Tamburino C. (2011). Antiplatelet and anticoagulant therapy in elderly patients. G. Ital. Cardiol. (2006).

[87] Laborde C., Barben J., Mihai A.M., Nuss V., Vovelle J., d’Athis P., Jouanny P., Putot A., Manckoundia P. (2020). Impact of Age, Multimorbidity and Frailty on the Prescription of Preventive Antiplatelet Therapy in Older Population. Int. J. Environ. Res. Public Health.

[88] Hylek E.M., Evans-Molina C., Shea C., Henault L.E., Regan S. (2007). Major hemorrhage and tolerability of warfarin in the first year of therapy among elderly patients with atrial fibrillation. Circulation.

[89] Mant J., Hobbs F.D., Fletcher K., Roalfe A., Fitzmaurice D., Lip G.Y., Murray E. (2007). Warfarin versus aspirin for stroke prevention in an elderly community population with atrial fibrillation (the Birmingham Atrial Fibrillation Treatment of the Aged Study, BAFTA): A randomised controlled trial. Lancet.

[90] Gurwitz J.H., Avorn J., Ross-Degnan D., Choodnovskiy I., Ansell J. (1992). Aging and the anticoagulant response to warfarin therapy. Ann. Intern. Med..

[91] Lucà F., Oliva F., Abrignani M.G., Di Fusco S.A., Parrini I., Canale M.L., Giubilato S., Cornara S., Nesti M., Rao C.M. (2023). Management of Patients Treated with Direct Oral Anticoagulants in Clinical Practice and Challenging Scenarios. J. Clin. Med..

[92] Barben J., Menu D., Rosay C., Vovelle J., Mihai A.M., Nuss V., d’Athis P., Putot A., Manckoundia P. (2020). The prescription of direct oral anticoagulants in the elderly: An observational study of 19 798 Ambulatory subjects. Int. J. Clin. Pract..

[93] Manckoundia P., Rosay C., Menu D., Nuss V., Mihai A.M., Vovelle J., Nuémi G., d’Athis P., Putot A., Barben J. (2020). The Prescription of Vitamin K Antagonists in a Very Old Population: A Cross-Sectional Study of 8696 Ambulatory Subjects Aged Over 85 Years. Int. J. Environ. Res. Public Health.

[94] Salih M., Abdel-Hafez O., Ibrahim R., Nair R. (2021). Atrial fibrillation in the elderly population: Challenges and management considerations. J. Arrhythm..

[95] Loirat A., Fénéon D., Behaghel A., Behar N., Le Helloco A., Mabo P., Daubert J.C., Leclercq C., Martins R.P. (2015). Pacemaker replacement in nonagenarians: Procedural safety and long-term follow-up. Arch. Cardiovasc. Dis..

[96] Fauchier L., Alonso C., Anselme F., Blangy H., Bordachar P., Boveda S., Clementy N., Defaye P., Deharo J.C., Friocourt P. (2016). Position paper for management of elderly patients with pacemakers and implantable cardiac defibrillators: Groupe de Rythmologie et Stimulation Cardiaque de la Société Française de Cardiologie and Société Française de Gériatrie et Gérontologie. Arch. Cardiovasc. Dis..

[97] Kong M.H., Al-Khatib S.M., Sanders G.D., Hasselblad V., Peterson E.D. (2011). Use of implantable cardioverter-defibrillators for primary prevention in older patients: A systematic literature review and meta-analysis. Cardiol. J..

[98] Fauchier L., Marijon E., Defaye P., Piot O., Sadoul N., Perier M.C., Gras D., Klug D., Algalarrondo V., Bordachar P. (2015). Effect of age on survival and causes of death after primary prevention implantable cardioverter-defibrillator implantation. Am. J. Cardiol..

[99] Moss A.J., Zareba W., Hall W.J., Klein H., Wilber D.J., Cannom D.S., Daubert J.P., Higgins S.L., Brown M.W., Andrews M.L. (2002). Prophylactic implantation of a defibrillator in patients with myocardial infarction and reduced ejection fraction. N. Engl. J. Med..

[100] Bardy G.H., Lee K.L., Mark D.B., Poole J.E., Packer D.L., Boineau R., Domanski M., Troutman C., Anderson J., Johnson G. (2005). Amiodarone or an implantable cardioverter-defibrillator for congestive heart failure. N. Engl. J. Med..

[101] Tsai V., Goldstein M.K., Hsia H.H., Wang Y., Curtis J., Heidenreich P.A. (2011). Influence of age on perioperative complications among patients undergoing implantable cardioverter-defibrillators for primary prevention in the United States. Circ. Cardiovasc. Qual. Outcomes.

[102] Scheurlen C., van den Bruck J., Wörmann J., Plenge T., Sultan A., Steven D., Lüker J. (2021). ICD therapy in the elderly: A retrospective single-center analysis of mortality. Herzschrittmachertherapie + Elektrophysiologie.

[103] Malekrah A., Shafiee A., Heidari A., Vasheghani-Farahani A., Bozorgi A., Sadeghian S., Yaminisharif A. (2023). Predictors of mortality and clinical outcomes following implantable cardioverter-defibrillator therapy in elderly patients: A retrospective single-center cohort study. Health Sci. Rep..

[104] Zakine C., Garcia R., Narayanan K., Gandjbakhch E., Algalarrondo V., Lellouche N., Perier M.C., Fauchier L., Gras D., Bordachar P. (2019). Prophylactic implantable cardioverter-defibrillator in the very elderly. Europace.

[105] Haines D.E., Wang Y., Curtis J. (2011). Implantable cardioverter-defibrillator registry risk score models for acute procedural complications or death after implantable cardioverter-defibrillator implantation. Circulation.

[106] Russo A.M., Stainback R.F., Bailey S.R., Epstein A.E., Heidenreich P.A., Jessup M., Kapa S., Kremers M.S., Lindsay B.D., Stevenson L.W. (2013). ACCF/HRS/AHA/ASE/HFSA/SCAI/SCCT/SCMR 2013 appropriate use criteria for implantable cardioverter-defibrillators and cardiac resynchronization therapy: A report of the American College of Cardiology Foundation appropriate use criteria task force, Heart Rhythm Society, American Heart Association, American Society of Echocardiography, Heart Failure Society of America, Society for Cardiovascular Angiography and Interventions, Society of Cardiovascular Computed Tomography, and Society for Cardiovascular Magnetic Resonance. J. Am. Coll. Cardiol..

[107] Zeppenfeld K., Tfelt-Hansen J., de Riva M., Winkel B.G., Behr E.R., Blom N.A., Charron P., Corrado D., Dagres N., de Chillou C. (2022). 2022 ESC Guidelines for the management of patients with ventricular arrhythmias and the prevention of sudden cardiac death. Eur. Heart J..

[108] Aso S., Ono S., Michihata N., Uemura K., Yasunaga H. (2023). Effectiveness of vaccination on influenza-related critical illnesses in the elderly population. J. Infect. Chemother..

[109] Scherrenberg M., Zeymer U., Schneider S., Van der Velde A.E., Wilhelm M., Van’t Hof A.W.J., Kolkman E., Prins L.F., Prescott E., Iliou M.C. (2021). EU-CaRE study: Could exercise-based cardiac telerehabilitation also be cost-effective in elderly?. Int. J. Cardiol..

[110] Cuesta-Vargas A.I., Fuentes-Abolafio I.J., García-Conejo C., Díaz-Balboa E., Trinidad-Fernández M., Gutiérrez-Sánchez D., Escriche-Escuder A., Cobos-Palacios L., López-Sampalo A., Pérez-Ruíz J.M. (2023). Effectiveness of a cardiac rehabilitation program on biomechanical, imaging, and physiological biomarkers in elderly patients with heart failure with preserved ejection fraction (HFpEF): FUNNEL + study protocol. BMC Cardiovasc. Disord..

[111] Akinmoladun O.F., Femi F.A., Nesamvuni C.N. (2022). Implication of knowledge, lifestyle and self-efficacy in the prevention of cardiovascular diseases’ risk factors among the urban elderly. Nutr. Health.

[112] Vilela de Sousa T., Cavalcante A., Lima N.X., Souza J.S., Sousa A.L.L., Brasil V.V., Vieira F.V.M., Guimarães J.V., de Matos M.A., Silveira E.A. (2023). Cardiovascular risk factors in the elderly: A 10-year follow-up survival analysis. Eur. J. Cardiovasc. Nurs..

[113] Trtica Majnarić L., Bosnić Z., Kurevija T., Wittlinger T. (2021). Cardiovascular risk and aging: The need for a more comprehensive understanding. J. Geriatr. Cardiol..

[114] Noale M., Limongi F., Maggi S. (2020). Epidemiology of Cardiovascular Diseases in the Elderly. Adv. Exp. Med. Biol..

[115] Williams B., Mancia G., Spiering W., Agabiti Rosei E., Azizi M., Burnier M., Clement D.L., Coca A., de Simone G., Dominiczak A. (2018). 2018 ESC/ESH Guidelines for the management of arterial hypertension. Eur. Heart J..

[116] (2019). Efficacy and safety of statin therapy in older people: A meta-analysis of individual participant data from 28 randomised controlled trials. Lancet.

[117] González-Colaço Harmand M., García-Sanz M.D.M., Agustí A., Prada-Arrondo P.C., Domínguez-Rodríguez A., Grandal-Leirós B., Peña-Otero D., Negrín-Mena N., López-Hernández J.J., Díez-Villanueva P. (2022). Review on the management of cardiovascular risk factors in the elderly. J. Geriatr. Cardiol..

